# Post-stroke kinematic analysis in rats reveals similar reaching abnormalities as humans

**DOI:** 10.1038/s41598-018-27101-0

**Published:** 2018-06-07

**Authors:** Gustavo Balbinot, Clarissa Pedrini Schuch, Matthew S. Jeffers, Matthew W. McDonald, Jessica M. Livingston-Thomas, Dale Corbett

**Affiliations:** 1 Department of Cellular and Molecular Medicine, Faculty of Medicine, University of Ottawa, Ottawa, ON Canada; 2 Canadian Partnership for Stroke Recovery, University of Ottawa, Ottawa, ON Canada; 3 Brain Institute, Federal University of Rio Grande do Norte, Natal, RN Brazil

## Abstract

A coordinated pattern of multi-muscle activation is essential to produce efficient reaching trajectories. Disruption of these coordinated activation patterns, termed synergies, is evident following stroke and results in reaching deficits; however, preclinical investigation of this phenomenon has been largely ignored. Furthermore, traditional outcome measures of post-stroke performance seldom distinguish between impairment restitution and compensatory movement strategies. We sought to address this by using kinematic analysis to characterize reaching movements and kinematic synergies of rats performing the Montoya staircase task, before and after ischemic stroke. Synergy was defined as the simultaneous movement of the wrist and other proximal forelimb joints (i.e. shoulder, elbow) during reaching. Following stroke, rats exhibited less individuation between joints, moving the affected limb more as a unit. Moreover, abnormal flexor synergy characterized by concurrent elbow flexion, shoulder adduction, and external rotation was evident. These abnormalities ultimately led to inefficient and unstable reaching trajectories, and decreased reaching performance (pellets retrieved). The observed reaching abnormalities in this preclinical stroke model are similar to those classically observed in humans. This highlights the potential of kinematic analysis to better align preclinical and clinical outcome measures, which is essential for developing future rehabilitation strategies following stroke.

## Introduction

In order to stabilize movement trajectories it is necessary to coordinate movements between limb segments and joints^1–4^. Temporal coordination of these between-joint movements is termed, “kinematic synergy”, and represents the building blocks used to construct a variety of complex behaviours^5,6^. In humans, under normal circumstances, coordination of multiple limb segments relies on concurrent activation of elbow extensors and inactivation of elbow flexors to accurately and efficiently reach for a target. In this way, kinematic synergies relate to muscular synergies, in that coordinated movement of the joints rely on coordinated activation of the appropriate muscle groups^4^. However, kinematic synergies are much easier to reliably obtain than direct measurement of muscular synergy during reaching tasks in rodents, due to the small size of these animals^7^.

Stroke often disrupts synergies due to a loss of cortical/subcortical input to muscles and altered reflex activity, contributing to impaired movements and coordination^2,8,9^. Following stroke, abnormal flexor synergy is evident during reaching and is characterized by excessive elbow flexion, shoulder abduction, and internal rotation, resulting in an inability of the stroke patient to efficiently perform task-related movements^3,10^. Normally, voluntary reaching is also accompanied by a number of individualized movements between joints; however, after a stroke this individualization is reduced, resulting in the limb moving as more of a single unit^8,11^. Emergence of these abnormal movement synergies following stroke^9,12,13^ compromises the ability to independently perform activities of daily living^14^ and reduces the quality of life of stroke survivors^15^.

Clinical research often describes abnormal movement synergies using indices such as the Fugl-Meyer scale^16^ or kinematic analysis^5,17,18^. To date, analogous evidence of abnormal synergies in rodents are lacking in preclinical studies. Preclinical research has typically used performance-based measures such as single pellet reaching or the Montoya staircase to quantify forelimb reaching impairments and assess recovery of function following stroke^19–21^. These tests only measure the number of food pellets retrieved (a performance index), and lack any metric of the kinematics of the movement used to perform the pellet retrieval. While these outcome tests have merit, they assess only success or failure to retrieve a pellet, and do not take differences in movement synergies into account. Although extensive analysis of rodent movement patterns has been performed using categorical scales such as Eshkol-Wachmann movement notation^22–28^, quantitative evaluation of more discrete movements has been limited^29,30^. Thus, in order to facilitate the examination of pharmacological or rehabilitative interventions in rodent models following stroke, and improve the translation of these findings to the clinic, a more detailed quantification of rodent reaching patterns and movement synergies is warranted. This is important since post-stroke synergies are strongly associated with functional outcomes and predictors of recovery^31–33^. The present study used kinematic analysis in the Montoya staircase task to quantify and compare forelimb reaching movement and kinematic synergies in rats before and after ischemic stroke. We hypothesized that deficits in reaching performance following stroke would be accompained by abnormal movement synergies, and that the reaching abnormalities observed in rats would be similar to those observered in humans following stroke.

## Results

**Figure 1:**
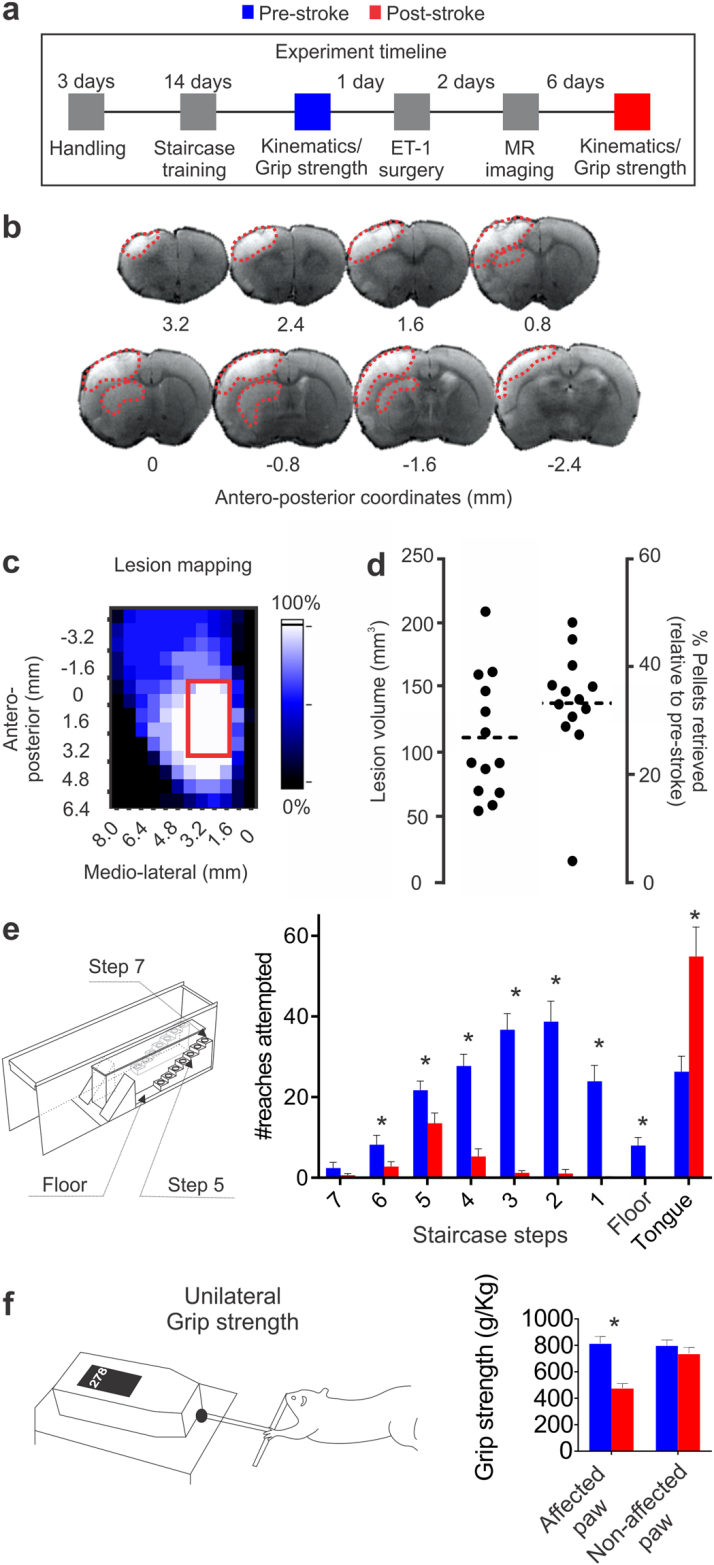
Experimental timeline, ischemic lesion, and performance on the staircase task. (**a**) Experimental timeline. (**b**) MRI images depict a representative ischemic lesion (~115.0 mm^3^); the lighter coloured area in each coronal section is the ischemic region (red dashed lines). (**c**) Ischemic lesions were predominantly located in the caudal forelimb area in all animals (red rectangle)^36^. Voxel colour indicates proportion (0–100%) of animals with a lesion at a given location. (d, left panel) Lesion volume and (d, right panel) staircase impairment 8 days post-stroke. Each dot represents an individual animal, with the dotted line representing the mean of all animals. (e, left panel) Depicts staircase task and (e, right panel) number of reaches attempted to each staircase step. Following stroke animals made fewer reach attempts to most staircase steps. (**f**) Grip strength of the affected paw was reduced following stroke. *pre- versus post-stroke (p < 0.05). Paired t-test, n = 13 and mean ± s.e.m. for this and all subsequent figures.

To assess post-stroke changes in forelimb movement, a within-subjects crossover design was used, wherein reaching kinematics were analyzed in the same rats both pre- and 8 days post-stroke (N = 13; Fig. 1a).

### Forelimb motor cortex stroke produces reaching impairments

To induce focal stroke in the rat forelimb sensorimotor cortex and dorso-lateral striatum, we used intra-cerebral endothelin-1 (ET-1) injections^34^ and measured infarct volume using T2-weighted MRI (Fig. 1b). Lesions encompassed most of the forelimb area, dorsolateral striatum, and infringed upon adjacent cortical areas (Fig. 1c)^35,36^.

Significant reaching impairments (paired t-test, p < 0.001; Fig. 1d) were evident 8 days following stroke on the staircase task. Video analysis showed that after stroke the number of reach attempts to most staircase steps was reduced, and the number of attempts to retrieve a pellet with the tongue increased (paired t-test and stepwise Holm-Bonferroni corrections, p = 0.013 for step 6, p = 0.030 for step 5, p < 0.001 for steps 4, 3, 2 and 1, p = 0.002 for floor, and p = 0.001 for tongue attempts; Fig. 1e). Additionally, the number of tongue attempts was positively correlated with the reaching impairment (Pearson correlation, r = 0.65, p = 0.017). Step 5 was chosen for further kinematic analysis since animals showed the most reach attempts to this step and cannot reach this step with their tongue (personal observation). Reduced grip strength was also evident following stroke (paired t-test, p < 0.001; Fig. 1f).

### Kinematic analysis of reaching behavior

**Figure 2:**
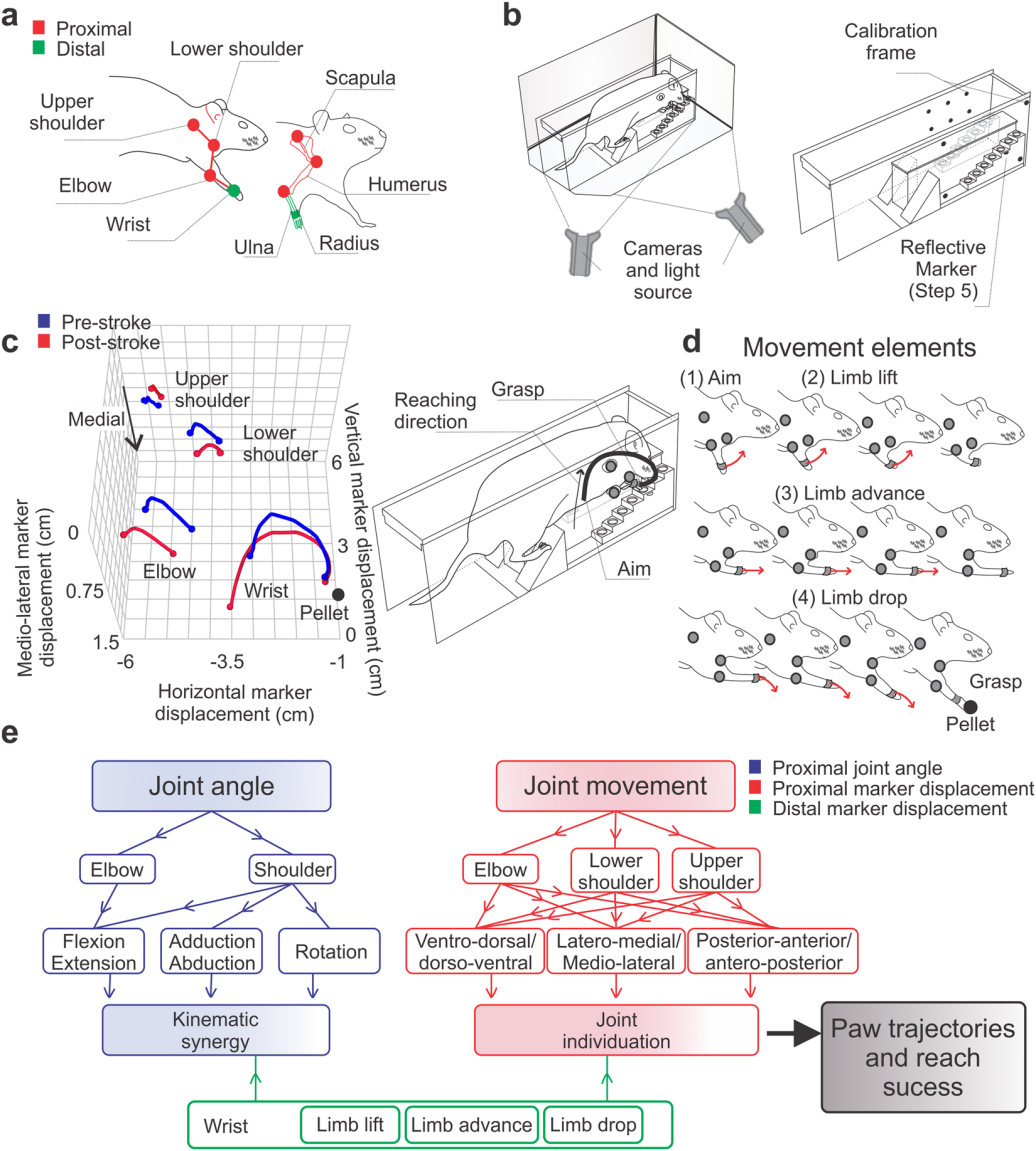
Proximal and distal upper limb movements measured using kinematic analysis. (**a**) Location of reflective markers (red: proximal segments; green: distal segments). **(b)** Two synchronized video cameras were used to determine the coordinates of upper limb movements. A calibration frame and a reference marker (step 5) were used to calibrate and normalize the kinematic data. (c, left panel) Reconstruction showing the mean reaching trajectories in the staircase (blue: pre-stroke, red: post-stroke). The black trace (c, right panel) shows the wrist movement during post-stroke reaching. (**d**) Reaching was divided into four movement elements^23,37^: (1) aim; a static position of the limb used to aim the paw before the beginning of the reaching movement, (2) limb lift; the upward paw movement that precedes limb advance, (3) limb advance; the forward movement of the paw towards the pellet, and (4) limb drop; the downward movement of the paw to reach for the pellet. (**e**) Summary of terminology and components of synergy analysis used throughout the manuscript. Proximal elbow (flexion/extension) and shoulder joint (flexion/extension, adduction/abduction and internal/external rotation) angles (blue) or movements (red) were compared to distal wrist movements (green) to compute kinematic synergy and joint individuation. When attempting a voluntary reaching movement, the proximal upper limb segments (elbow and shoulder) work in coordination to stabilize wrist trajectory and to facilitate reaching and grasping. Abnormalities in kinematic synergies and joint individuation have been associated with muscle spasticity (kinematic synergy)^5,31,32^ and impairments in coordination (joint individuation)^11,38^ possibly leading to unstable wrist trajectories and reduced reach success (black arrow and box).

**Figure 3:**
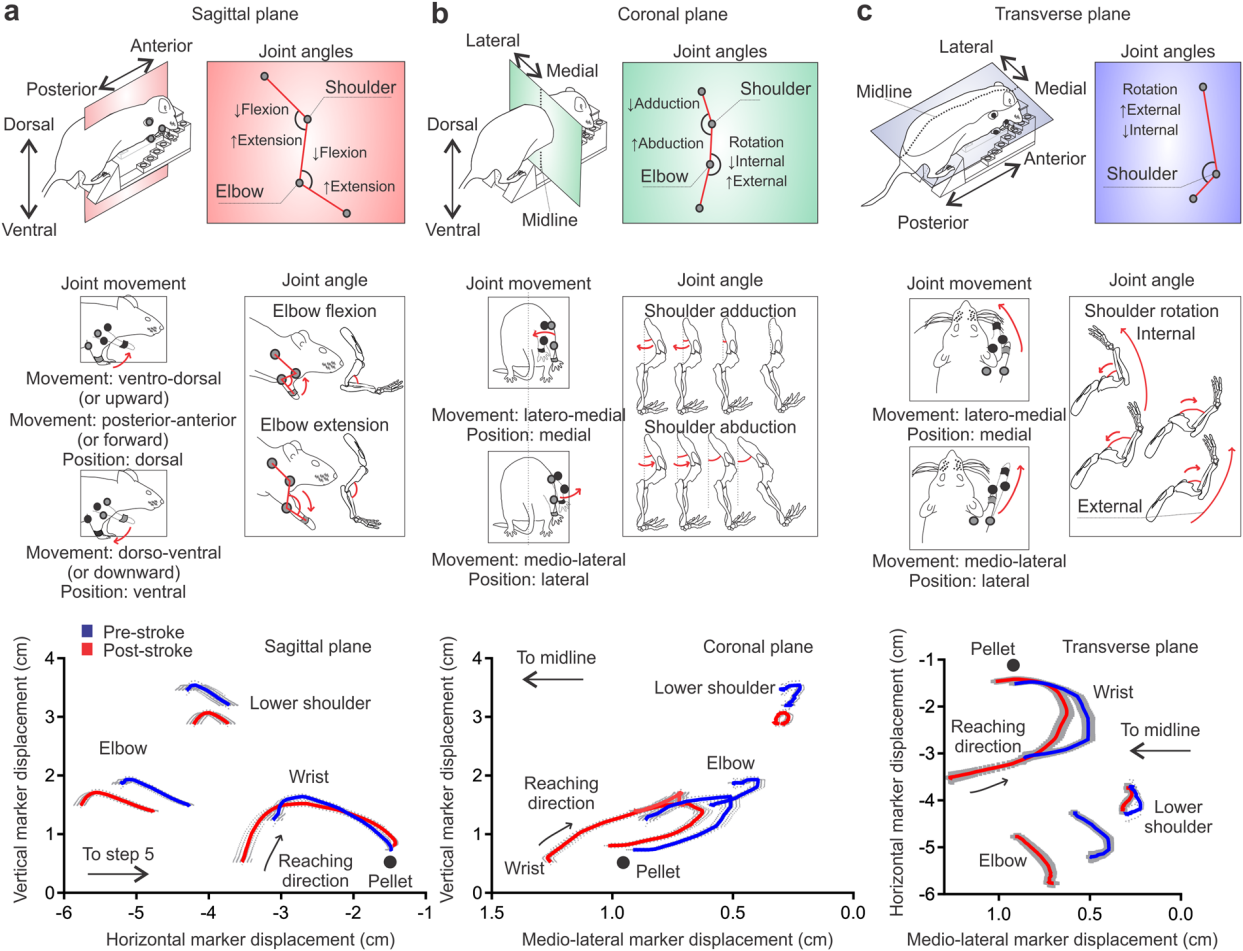
Summary of terminology for joint movements and angles. (**a**) Joint movement in the sagittal plane is ventro-dorsal (upward), dorso-ventral (downward), posterior-anterior (forward) and anterior-posterior (backward). The angular motion of the elbow and shoulder joints in the sagittal plane is flexion or extension. (**b**) Joint movement in the coronal plane is latero-medial or medio-lateral. The angular motion of the shoulder joint in the coronal plane is abduction or adduction. (**c**) Joint movement in the transverse plane is latero-medial or medio-lateral. The angular motion of the shoulder joint in the coronal plane is external or internal rotation, and is measured using elbow joint angle (see methods). Bottom panels of (**a**–**c**) show the marker displacement of the wrist, elbow and shoulder joints in the sagittal, coronal, and transverse planes, respectively. Mean ± s.e.m. traces (n = 13).

We measured the coordinates of an upper limb kinematic model using reflective markers positioned at the wrist, elbow, lower and upper shoulder (Fig. 2a–d). Unless specifically outlined, kinematic synergy (angle) or joint individuation (marker displacement) was defined as the simultaneous movement of proximal (i.e., elbow, lower and upper shoulder) and distal (i.e., wrist) joints (Fig. 2e). Description of joint angles, joint movements, planes of motion and forelimb displacement to the target are provided in Fig. 3.

Following stroke, animals show a less upright posture (Fig. 3a, lower panel); indicated by more ventral positioning of the lower shoulder, wrist, and elbow joints throughout the reaching cycle. These joints were also held more closely to the midline throughout reaching (Fig. 3b,c, lower panels). Supplementary Figure S1 provides detailed results and statistical comparisons of pre- and post-stroke changes in joint position.

### Emergence of abnormal elbow flexion, shoulder adduction and rotation kinematic synergies following stroke

**Figure 4:**
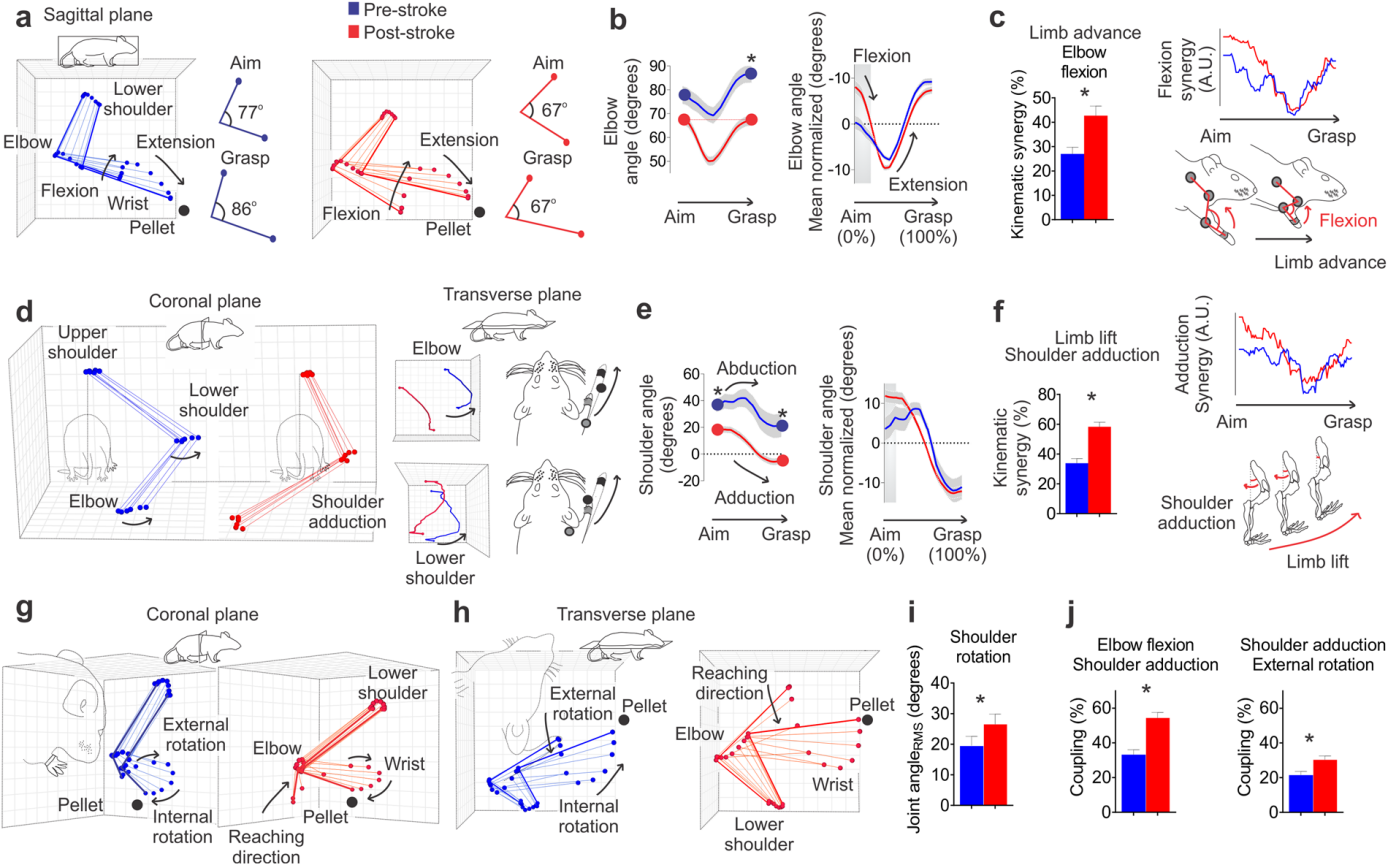
Abnormal elbow flexion, shoulder adduction and rotation kinematic synergy following stroke. (**a**) Representation of elbow angles in the sagittal plane throughout the reach. Elbow flexion is used to lift the paw before advancing the limb toward the target and elbow extension is used to extend the paw towards the pellet (black arrows). (b, left panel) Animals displayed greater elbow extension at grasp position relative to aim position pre-stroke; however, following stroke aim and grasp angles are similar. (b, right panel) During the first segment of the reach, animals show greater elbow flexion. (**c**) Prior to stroke, elbow movement involves increased extension during limb advance (decreased elbow flexion synergy); however after stroke animals display increased elbow flexion throughout limb advance (increased elbow flexion synergy; left panel). Further, the increased flexion synergy occurred mainly during the first half of the reaching movement (right panels). (**d**) Pre-stroke reaching involves medio-lateral elbow and lower shoulder movement to facilitate pronation of the limb during the reach. Black arrows indicate these elbow and shoulder movements in both the coronal (left panel) and transverse planes (right panels). (e, left panel) Following stroke the shoulder is more adducted during the aim and grasp. Pre-stroke movement uses shoulder abduction to transport the limb, while post-stroke movement displays adduction only (black arrows). (e, right panel) Specifically, animals showed increased shoulder adduction during the beginning of the reach. (**f**) Post-stroke animals display increased shoulder adduction synergy during limb lift (left panel) that was more evident during the first half of the reaching movement (right panels). (**g**–**i**) Shoulder rotation movements were increased following stroke, and were characterized by increased shoulder external and internal rotation angles (shown in two different views: coronal, **g**; and transverse, **h**). (**i**) Root mean square of shoulder internal/external rotation angle. (**j**) Abnormal coupling between elbow flexion/shoulder adduction and shoulder adduction/external rotation were evident post-stroke. Mean traces and stick figures (n = 13). Paired t-test, ^*^p < 0.05, mean, or mean ± s.e.m.

Pre-stroke, when transitioning from aiming to grasping, the elbow initially flexes and then extends beyond the initial aiming angle. Post-stroke, the elbow is more flexed throughout the reach and does not extend beyond the initial angle (paired t-test, p = 0.008; Fig. 4a,b). During the beginning stages of the reach (1–22% of total reach), animals showed increased elbow flexion following stroke (Bonferroni-corrected post-hoc test, p < 0.001; Fig. 4b, right panel). This is likely associated with the emergence of an abnormal flexor synergy during limb advance (paired t-test, p < 0.001; Fig. 4c and Supplementary Video 1). Multiple linear regression showed that grip strength was positively correlated with elbow flexion synergy (R = 0.649; p = 0.016), indicating that this synergy was used adaptatively in rats with less loss of strength (Supplementary Table 1).

Since shoulder movements are typically used to facilitate forepaw pronation and allow successful pellet grasping^19,23,37^, we investigated how elbow and shoulder medio-lateral movements changed following stroke. Post-stroke, animals displayed a reduced ability to abduct the shoulder joint (i.e., move the elbow and lower shoulder laterally; Fig. 4d); in fact, animals utilized shoulder adduction (the opposite movement) during the initial stages of the reach (1–12% of total reach; Bonferroni-corrected post-hoc test, p < 0.001; Fig. 4e, right panel). Pre-stroke, the shoulder was more abducted at the aim position, moved more laterally throughout the first half of reaching (paired t-test, p = 0.002), and ended significantly more abducted at the final grasp position compared to post-stroke (paired t-test, p = 0.005; Fig. 4e). Conversely, post-stroke movement displayed abnormal adduction kinematic synergy with the shoulder joint during limb lift compared to pre-stroke (paired t-test, p < 0.001; Fig. 4f). Further analysis demonstrated a significant negative correlation between lesion size and the ability to abduct the shoulder during limb advance (Pearson correlation, r = −0.64, p = 0.018, Supplementary Table 2, see shoulder angle in the transverse plane), as such, larger lesions were associated with more abnormal adduction synergy.

Post-stroke movements also showed increased internal/external shoulder rotation throughout the reach (defined as root mean square of joint angle; paired t-test, p = 0.048; Fig. 4g–i). These increased rotation movements at the shoulder were positively correlated with lesion volume following stroke (Pearson correlation, r = 0.64, p = 0.017, Supplementary Table 2, see elbow angle in the coronal plane). The percentage of kinematic synergy between shoulder internal rotation during limb advance was positively associated with lesion size (Pearson correlation, r = 0.67, p = 0.011, Supplementary Table 2, see elbow angle in the coronal plane). To investigate the occurrence of simultaneous kinematic synergies a coupling index was calculated (see Methods). The coupling index compares the angular variation in the elbow and shoulder joints, the index is a measure of simultaneous angular movement between elbow (flexion, extension) and shoulder (rotations, abduction and adduction). This analysis indicated that elbow flexion-shoulder adduction (paired t-test, p < 0.001) and shoulder adduction-external rotation occurred concurrently (paired t-test, p = 0.008; Fig. 4j).

Multiple linear regression showed that the proportion of pellets retrieved post-stroke in relation to pre-stroke was negatively correlated with the coupling of shoulder adduction and external rotation (R = −0.627, p = 0.022; Supplementary Table 1), such that rats with a higher level of coupling performed worse in the staircase task.

### Post-stroke proximal movements are less individuated as a result of excessive flexion during limb advance

**Figure 5:**
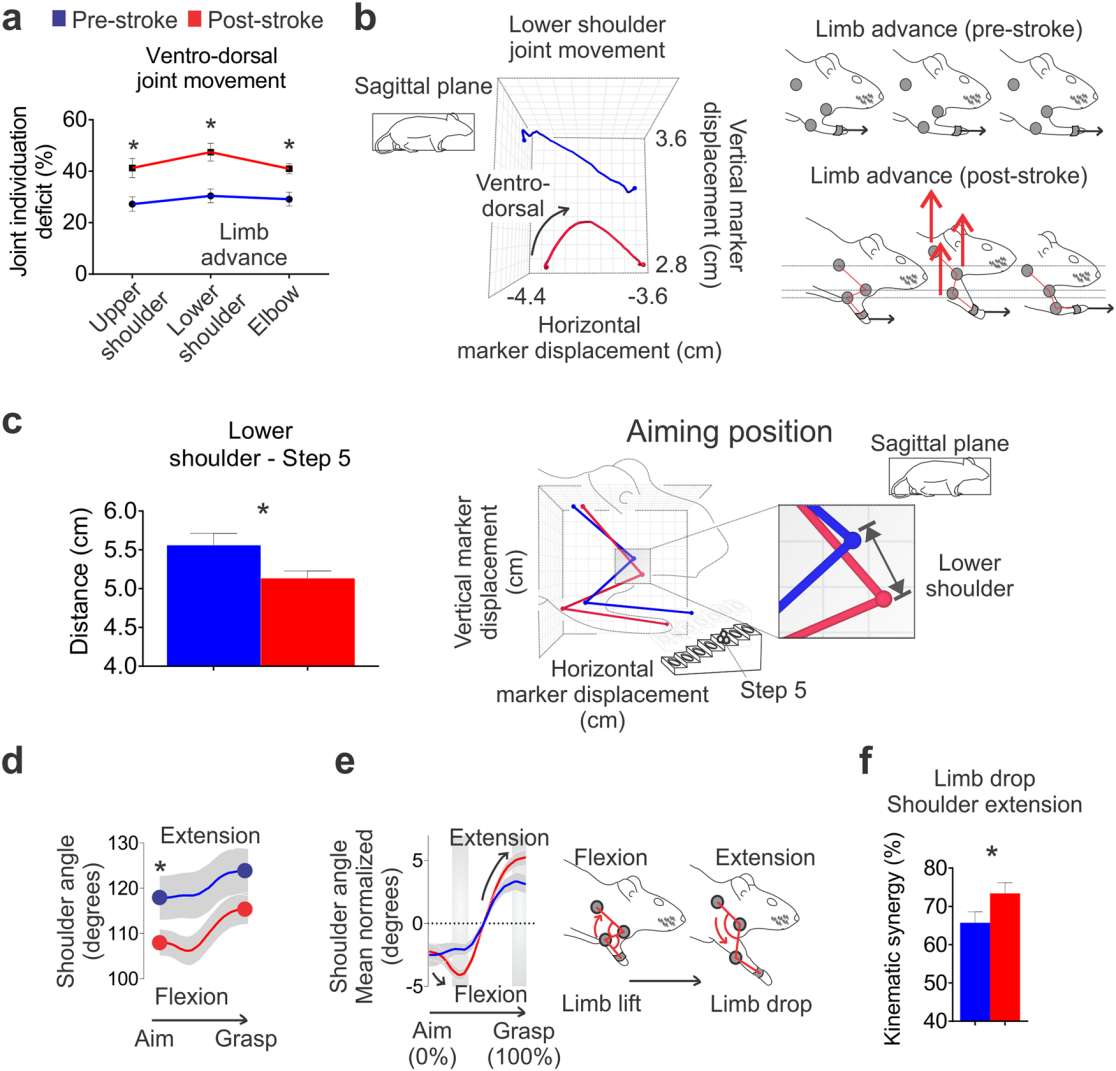
Post-stroke reaching displays less individuated movements and more shoulder extension. (**a**) Excessive elbow and shoulder ventro-dorsal movements were evident during limb advance following stroke. (**b**) An example of increased lower shoulder ventro-dorsal movements observed following stroke (black arrow in the left panel). A schematic illustration of these movement abnormalities (red arrows in the right panel). (**c**) Following stroke, the lower shoulder was postitioned closer to the target (step 5) during the initial aiming position. (**d**) Post-stroke, the shoulder was more flexed while at the aiming position. (**e**) Increased shoulder flexion and extension was evident following stroke during the initial and final stages of reaching, respectively (black arrows in the left panel); visual representation of the increased shoulder flexion during limb lift and shoulder extension during limb drop (right panel). (**f**) During limb drop, there was increased shoulder extension synergy post-stroke. Mean traces and stick figures (n = 13). Paired t-test, ^*^p < 0.05. Linear mixed effects modeling and Bonferroni-corrected post-hoc tests, shaded areas p < 0.05, mean, or mean ± s.e.m.

Post-stroke movement was characterized by less individuated movements, such as the use of excessive and simultaneous ventro-dorsal movements at all proximal segments during limb advance (paired t-test, elbow, p = 0.008; lower shoulder, p = 0.001; and upper shoulder p = 0.011; Fig. 5a,b). During limb lift, this abnormal joint individuation of proximal joints was positively correlated with lesion volume (Pearson correlation, r = 0.57, p = 0.039; Supplementary Table 2, see lower shoulder position in the ventro-dorsal axis).

After stroke, animals positioned the shoulder joint closer (~0.5 cm) to the target step (paired t-test, p = 0.003; Fig. 5c). Post-stroke movement was characterized by more shoulder flexion during paw aiming (paired t-test, p = 0.030; Fig. 5d) and more shoulder extension during limb drop to grasp the pellet (Fig. 5e,f). Similarly, this shoulder movement pattern differed pre- and post-stroke during the initial stages of the reach (25–40% of total reach; increased shoulder flexion) and towards the end of the reach (87–100% of total reach; increased shoulder extension; Bonferroni-corrected post-hoc test, p < 0.001; Fig. 5e). Shoulder extension kinematic synergy was increased during limb drop post-stroke (paired t-test, p = 0.047; Fig. 5f).

### Inefficient wrist trajectories following stroke

**Figure 6:**
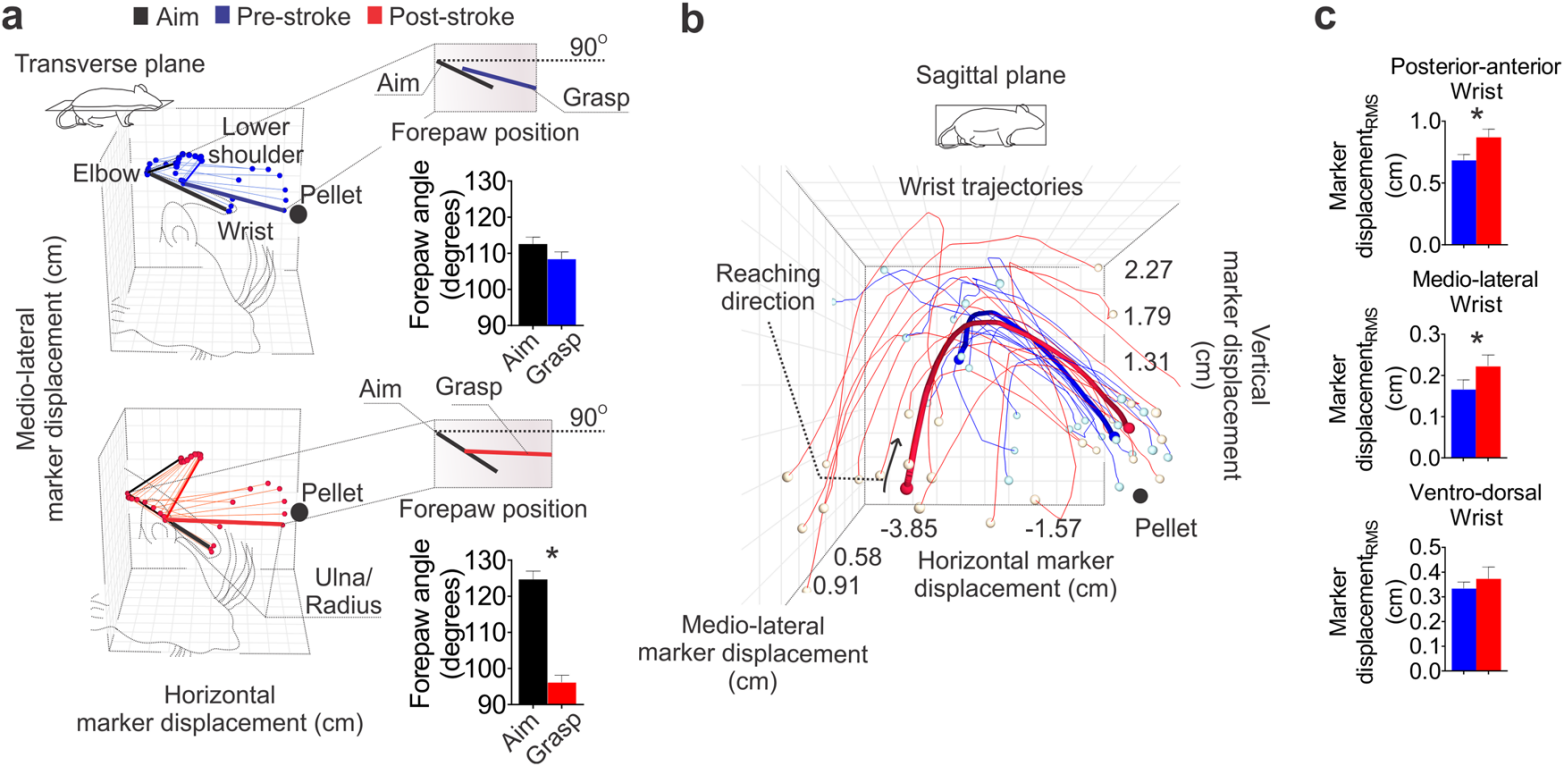
Abnormal wrist trajectories following stroke. (**a**) Pre-stroke aiming position closely resembles final grasping position, while post-stroke aiming and grasping positions are different (aim position: black line grasp position: colored line). (**b**,**c**) There was increased posterior-anterior and medio-lateral wrist joint movements, while no differences were apparent in the ventro-dorsal plane. Mean traces (thick lines) and individual traces (thin lines). Mean stick figures (n = 13). Paired t-test, ^*^p < 0.05, mean ± s.e.m.

An important consideration of our study was whether alterations in proximal limb movement (shoulder/elbow) affect the trajectory of the most-distal segments (paw/wrist). An overview of pre- and post-stroke forelimb position and trajectory shows changes of initial aiming position and reaching trajectory between pre- and post-stroke movements (paired t-test, p < 0.001; Fig. 6a). Although not statistically different, the number of velocity adjustments during vertical (pre = 2.07 ± 0.14 and post = 2.43 ± 0.17) and medio-lateral (pre = 2.65 ± 0.22 and post = 3.33 ± 0.26) movements appeared to increase post-stroke (paired t-test, p = 0.098 and p = 0.068, respectively). Greater joint movement in the posterior-anterior and medio-lateral planes was evident (paired t-test, p = 0.010 and p = 0.034, respectively) with no changes in joint movement in the ventro-dorsal plane (paired t-test, p > 0.05; Fig. 6b,c). This led to a significant increase in path length to the target (pre = 4.06 cm ± 0.13 cm and post = 4.41 cm ± 0.14 cm; paired t-test, p = 0.035).

## Discussion

In the present study we were interested in determining whether rats have abnormalities in kinematic synergy following stroke and if so, whether the abnormalities were comparable to those observed in humans, as recent evidence suggests that rat and human recovery may follow similar patterns^39,40^. As previously mentioned, kinematic synergies are closely related to muscle synergies^5,6^, which are methodologically challenging to obtain in rodents during reaching tasks due to their small size^7^. Here, we report three abnormal kinematic synergies in rats during reaching following stroke: excessive elbow flexion, shoulder adduction, and shoulder external rotation. The severity of these synergies was also closely related to stroke volume, and their presence was predictive of the degree of impairment in grip strength and pellet retrieval. Furthermore, rats displayed increased use of ventro-dorsal shoulder and elbow “lifting” movements during reaching, and a reduction in trunk distance from the target prior to reaching. In many ways, this coupling of stroke-induced synergies was reminiscient of the classic flexor synergy, and compensatory adjustments to trunk position, that are commonly observed in humans following stroke^5,41^.

When reaching to a target following stroke, humans commonly demonstrate impairments in the same planes of joint motion that we identified in rats. For example, using a similar set of reflective markers (i.e. shoulder, elbow and wrist) and kinematic model as the current study, Levin *et al*. (2016) revealed that upper-limb flexor synergy in humans is used adaptively during post-stroke reaching, in a manner that is correlated to stroke severity^5^. In humans, abnormal coupling between upper-limb flexion and shoulder abduction/internal rotation has also been extensively described in the literature^9,13,16,31,32,42^. It is hypothesized that to overcome increased upper-limb flexor tonus, patients use excessive trunk movements to increase their degrees of freedom and position themselves closer to the target^43^. These examples clearly demonstrate that stroke in humans displays similar kinematic abnormalities as in rats, as observed in the present study. However, the composition of flexor synergy observed in rats differs somewhat from the “classic flexor synergy” observed in humans.

In humans, an abnormal coupling between elbow flexion and shoulder abduction/internal rotation commonly occurs during reaching movements following stroke^5,10,44^. In the rat, we found that elbow flexion was coupled to shoulder adduction and external rotation. This discrepancy is likely explained by differences in the shoulder joints between species. Rats are quadrupeds, therefore the forelimbs function to provide support for the trunk^45,46^. As such, the scapula is rotated into a lateral position, with a larger anterior area suggesting that muscles used in stabilization and rotation of the pectoral girdle are more developed than in bipedal species^47^. In addition, the narrower humerus at the epicondyles suggests less rotational capability and greater joint stability under loading^47^. This can change the expression of synergies. For example, abnormal co-contraction of shoulder muscles, such as *teres major*, results in shoulder adduction, rotation and flexion^7,48^, rather than the lateral scapular instability typically observed in humans (Supplementary Figure S2)^9,13,14^.

Regardless of these differences in flexor synergy between species, the ability to move the shoulder joint is strongly related to function of both the paw and hand^38,49^. Our results are consistent with previous reports of increased post-stroke wrist displacement and trajectory adjustments in mice^50^, rats^51^ and humans^43,52,53^. Human stroke recovery has been suggested to occur in a proximal to distal fashion, wherein proximal joint movements recover first to allow stabilization of further distal movements later^42^. Given that we observed a similar set of abnormal post-stroke movements between rats and humans, future studies should evaluate the time course at which different joints appear to recover their function.

For example, using the kinematic techniques described here, it is possible to investigate the potential impacts of motor map remodeling, rehabilitation-induced plasticity and pharmacological interventions on the emergence and control of abnormal synergies^1,6,54,55^. The use of more detailed outcome measures, such as kinematics, is an important consideration for preclinical studies since traditional performance-based measures of forelimb motor function do not capture the nuances between compensatory strategies and true recovery. Indeed, the lack of a correlation between pellet retrieval and reaching abnormalities reported in the present study demonstrates that overall performance on the Montoya staircase does not fully delineate stroke-induced impairments. Following stroke, the evaluation of forelimb motor function over time using these kinematic analyses could aid in distinguishing compensation versus recovery^41^. In humans, the emergence of abnormal synergies is related to lesion size and location^14^; this too is an area that has been largely understudied in preclinical research. Most experiments focus on small focal lesions to the forelimb motor cortex, whereas only a minority of human strokes are localized to this region^56^. Continuing to develop preclinical stroke models and outcome measures that have a strong concordance to human stroke is crucial to the successful translation of therapies from the bench to the bedside.

The use of sophisticated kinematic models in small laboratory animals is very challenging. The small size of rodent limbs and their proclivity to chew and remove joint markers limits the number of markers and amount of time available to obtain data within a reaching session. This leads to some limitations in the data that can be collected in rodents, compared to what would normally be used as the gold-standard in humans. For example, rotations would be better described using multiple rigid bodies placed on body segments and more complex mathematical approaches such as calculations of quartenions for rotation sequences^57^. However, placing the necessary number of markers on each limb segment, and restricting the rat from removing them was not feasible in the present study, leading us to use standard geometric algebra and a simpler kinematic model to estimate limb rotations. This means that data in the present study was calculated using 2D marker trajectories in 3 planes of movement (coronal, saggital, transverse), rather than a true 3D tracking of each marker. This could present a problem in the interpretation of some movements in a situation where the animal could freely reposition its body. However, we believe that due to the the restricted space of the staircase task creates a relatively fixed body position that limits out of plane movements, and the potential problems with interpretation of our data that may result. Caution should be taken if attempting to apply the same model used here to tasks where the subject can more freely change the positioning of its body, such as the single-pellet task.

In the present study, we used novel kinematic analyses to describe three abnormal kinematic synergies that emerge in rats following stroke: elbow flexion, shoulder adduction and shoulder rotation. Unlike humans, adult rodents have almost no direct afferents with spinal motoneurons^58^. Despite this, muscle synergies are likely to be organized intracortically, in both primates^54^ and rodents^59^ since intra-cortical microstimulation can evoke complex multijoint responses between species. Despite some differences in anatomy and central nervous system organization, stroke in rats results in abnormalities in muscle coordination similar to those observed in humans^5,41,44^, furthering the construct validity of using rats to study stroke impairment and recovery. International guidelines recommend using comparable preclinical and clinical outcome measures to minimize the potential of translational failure^20,60^. The present study demonstrates the feasibility of using kinematic analysis to align the methodology of impairment measurement between preclinical and clinical stroke studies. This approach will allow investigators to move beyond use of performance-based measures alone and thoroughly assess novel interventions in advance of being considered for translation to the clinical setting.

## Methods

### Experimental design

All procedures were approved by the University of Ottawa Animal Care Committee (protocol number: CMM-1816), in accordance with guidelines set by the Canadian Council on Animal Care. A total of 17 adult male Sprague Dawley rats weighing 280–350 g at the time of surgery were used.

The experimental timeline consisted of (i) 3 days of handling, (ii) 14 days of staircase training, (iii) pre-stroke kinematics acquisition, (iv) stroke lesion and (v) post-stroke retesting from days 5–8 post-stroke (from days 5–7 for performance measurments and day 8 for post-stroke kinematics acquisition) as we have done previously^34,61^. The 5–8 day time point was used given the evidence that very early (<5 days from stroke onset) retesting may lead to increased damage^61^. Animals without significant behavioral impairment (defined as >80% of training performance on staircase; N = 1) or those who refused to engage in post-stroke reaching (N = 1) were excluded from the analysis. A further two animals died during surgical procedures. Thus, data from a total of 13 animals were included in analyses.

Animals were housed in groups of 4 (plexiglas cages; 50 × 40 × 20 cm; L × W × H) on a reverse 12 hr light/dark cycle and all behavioral testing was performed during the dark phase. Animals were provided with food and water *ad libitum* except during behavioral training and testing, when they were food restricted to 85% body weight. An experimental timeline is shown in Fig. 1a.

### Stroke Surgeries

Stroke was induced in the hemisphere contra-lateral to the preferred paw, as previously described^34^. Briefly, under isoflurane anesthesia (4% induction, 2% maintenance) a midline scalp incision was made and three burr holes were drilled in the skull overlying the motor cortex and striatum. The vasoconstrictive peptide endothelin-1 (ET-1; 400 pmol/µl in sterile water, Abcam #120471, USA) was stereotaxically injected into the forelimb motor cortex (AP +2.3, ML +/−2.5, DV −1.7 and AP +0.0,+/−2.5, DV −1.7 mm from Bregma) and dorso-lateral striatum (AP +0.7, ML +/−3.8, DV −7.0). Following injection, the needle remained in place for 3 min to minimize backflow. For the duration of the surgery, body temperature was maintained between 36.5 °C and 37.5 °C using a heating pad.

### MR imaging

MR imaging was performed with a small animal magnetic resonance scanner (Agilent MR901 7 T, General Electric^®^, USA) in combination with a physiological monitoring system (SA instruments Inc., Stony Brook, USA) to monitor vital signs and maintain body temperature. Brain images were obtained with a T2-weighted fast spin echo pulse sequence with the following parameters: 15 axial (transverse) slices; slice thickness = 800 microns; in-plane resolution = 78 microns; echo train length = 8; echo time = 27 ms; scan time = 5 minutes. Lesion volumes were quantified based on MR images using ImageJ^®^ software (NIH, USA). Lesion maps were created using LabVIEW^®^ 8.5 routines (cortical lesion only); briefly, MR image voxels of cortical regions of interest (voxel size: 0.8 mm × 0.8mm) were considered as lesioned or non-lesioned (voxel colour indicating the proportion of animals with a lesion at a given location). While MR images can overestimate lesion size when collected soon-after stroke (48 hours) due to edema^62^, this measure positively correlates with histological infarct measures^62^.

### Montoya staircase skilled reaching

Skilled reaching was assessed using the Montoya staircase task^63^. Rats were placed into the staircase apparatus that consisted of a box and plinth. On either side of the plinth were 7 steps of increasing reaching difficulty, each baited with three sucrose pellets (45 mg; Test Diet^®^, USA). Two weeks before surgery, animals were trained (two 15 min trials per day for 14 days) to reach for pellets in the staircase task^63^. The number of pellets eaten per side was used as a measure of forelimb reaching success. Animals were tested before surgery to establish baseline performance and then retested for reaching ability 8 days after ischemic injury to determine level of impairment. Each test period consisted of 6 trials (2 trials per day for 3 days) with the last four trials used to assess reaching performance.

### Kinematic analysis

Detailed kinematic analysis was performed pre- and post-stroke as previously described^17^. Following the last day of staircase testing an additional Montoya staircase session was performed for kinematic analysis. Prior to filming, reflective markers were positioned on the upper and lower shoulder, elbow and wrist joints (Fig. 2). Anatomical reference points were located by an experienced evaluator to identify the wrist joint, elbow joint, proximal termination of the humerus (shoulder joint) and superior angle of the scapula (upper shoulder/trunk). Importantly, the upper shoulder and trunk movements described in the present study are intertwined (described as upper shoulder/trunk)^17^. By using a different set of reflective markers and kinematic models shoulder movements can be better characterized^17^. Montoya staircase reaching behavior does not have a pronounced retraction phase, like the hindlimb mid-stance and final contact phases during locomotion that can lead to inaccurate knee joint detection^64^. Therefore, the elbow marker was positioned by placing the rat forepaw in a protracted position (predominant during the reaching) to minimize skin movement artifacts^19,65^. Markers were made of wood beads (0.5 mm of diameter) wrapped by reflective tape (3 M^®^ ScotchliteTM, USA) and glued to the fur and skin using super glue (Loctite^®^, gel, Henkel Corp., USA). Sugar pellets were placed only on the side of the impaired limb and the reaching movement was recorded by two synchronized video cameras in the sagittal and dorsal view (Sony Handycam^®^, model HDR-PJ380, 60 fps, USA) for 8 minutes (Fig. 2b). Movement analysis of reaches included four movement elements^24,37^.

### Data analysis

All video files were analysed using Adobe Premier Pro^®^ software (Adobe Systems Inc., USA). Slow motion procedures were used to count all paw reaches and tongue extensions during each trial (8 minutes) and all videos of clear reaches to step 5 were used for kinematic analysis. The choice of step 5 for analysis is based on pilot studies that indicated that following stroke most of the reaches attempted are to this step. A clear reach was defined when: (1) the nose was directed toward the pellet (usually sniffing preceded reaching)^21^, (2) the animal was not supporting the body weight (with the reaching forepaw) using the lower stairs and (3) a clear ballistic movement was performed directly to step 5. When a reach was observed, the video was cropped from two video frames prior to initial movement detection until two video frames after the paw reached step 5. Reaches were not analyzed if there were multiple random attempts to reach pellets. An average of ≈6.4 reaches were analyzed per animal in each condition (i.e., pre- and post-stroke). Although only the reaches that met criteria were used for kinematic analysis, all reaches were counted for quantifying reaching attempts.

Reaching success was not considered in the kinematic analysis, only the reaching movement. Overall reaching success was measured by standard Montoya staircase analysis (number of pellets retrieved). All video data was digitized using open-source software (SkillSpector^®^ software, v. 1.3.2). Raw marker coordinates and joint angles were exported as an ASCII file. LabVIEW^®^ 8.5 custom software routines were developed to analyze kinematic data. Displacement data were normalized by step 5 coordinates, thus all displacement data is relative to the final endpoint of the reaching. For this, an additional reflective tape was attached to staircase step 5. Rotational movements of the elbow/shoulder complex were determined by differences in elbow joint angle^45^. Except where noted, shoulder external/internal rotation refers to elbow and shoulder complex external/internal rotation. We estimated the shoulder rotation (humeral rotation) using the relative angle between two vectors, ie. humerus (lower shoulder-elbow vector) and radio-ulna (elbow-wrist vector). Based in anatomical and functional descriptions of the rodent elbow joint we assume that the only way to change the radio-ulna segment (elbow-wrist vector) in relation to the shoulder (humerus segment, lower shoulder-elbow vector) in the coronal or transverse plane is using humeral rotation (internal-external). Only the shoulder joint have the degrees of freedom needed for this movement in these planes^45^. Thus, we estimated shoulder (humeral) rotation using the relative angle between the humerus (lower shoulder-elbow vector) and radio-ulna segments (elbow-wrist vector). Additionaly, angles in the coronal plane were intertwined flexion/extension-internal/external rotation (for elbow), and flexion/extension-abduction/adduction (for shoulder), referred to as pure internal/external rotation or abduction/adduction. Note that shoulder angles in the sagittal plane are also termed protraction/retraction angles^45,46^, herein termed as flexion/extension angles described elsewhere^17^.

### Position and angle

Position and angle data were interpolated to 100 frames, and represented 0 to 100% of the reaching cycle (i.e., from beginning to end of movement); mean subtracted to detect relative differences in signal patterning rather than absolute differences (i.e., signal variation is around zero) and filtered. Filtering was applied by averaging interpolated reaching events to avoid filtering artifacts on the signal edges. Position (marker displacement) and angle (joint angle) root mean square (RMS) was used to originate a single value that could describe pre- and post-stroke signal variation. Thus, the higher the variation of the signal, the higher the RMS value. Position RMS is termed as “marker displacement_RMS_” in cm and angle RMS as “joint angle_RMS_” in degrees.

Post- and pre-stroke joint movements and joint angles of each animal were subtracted and were used as inputs for linear regression and correlation analysis. Thus, negative values indicate decreased marker displacement or joint angle following stroke.

### Kinematic synergy and joint individuation

The relation between paw movements and angles was defined as ‘kinematic synergy’, paw movements and marker displacements as ‘joint individuation’^38^ and elbow and shoulder angles as ‘coupling index’. Kinematic synergy and joint individuation analysis between limb advance, limb lift and limb drop and other body segments movement changes was as follows. Wrist movement was detected and separated into 3 elements: (1) limb advance, (2) limb lift and (3) limb drop. For this the analysis was conducted using the first derivative of wrist antero-posterior (1), ventro-dorsal (2) and dorso-ventral (3) position signals to obtain the instantaneous variation of signals [Supplementary Figure S3; Equation (1)].

1 $${W}_{vel(x,y)}=\,\frac{d}{dt}[W{P}_{(x,y)}]$$

where, *W*_*vel(x*,*y)*_ is the wrist instantaneous velocity in the x (limb advance) or y (limb advance or limb drop) direction (cm.s^−1^), *WP*_*(x*,*y)*_ is the wrist relative position in the x or y direction (cm).

Whenever the wrist movements were detected by the algorithm [Equation (1)], they were categorized as limb advance, limb lift and limb drop based on derivative signals. Wrist movements were then divided into three different waveforms indicating the precise temporal location of the movement elements in relation to 0 to 100% of the reaching. Specifically, we identified limb advance using wrist posterior-anteror positive derivatives, limb lift using wrist ventro-dorsal positive derivatives and limb drop using wrist dorso-ventral negative derivatives. If there was no wrist or other body segment movement the frame was not counted by the algorithm. The initial and final frames were removed due to derivation artifacts and substituted for the subsequent/previous frame after applying Equations (1) and (2). The time stamps of limb advance, lift and drop were used to crop position (i.e., *BSP*_*(x*,*y*,*z)*_) and angle (*BJA*_*(x*,*y*,*z)*_) signals of other joints, that were also derivated to detect positive or negative changes based on the derivative signal [Equation (2)].

2 $$BS{V}_{(x,y,z)}=\,\frac{d}{dt}[BS{P}_{(x,y,z)}\,or\,BJ{A}_{(x,y,z)}]$$

where, *BSV*_*(x*,*y*,*z)*_ is the body segment instantaneous velocity (cm.s^−1^) or instantaneous angular velocity (degrees.s^−1^) in the x, y or z direction, *BSP*_*(x*,*y*,*z)*_ is the body segment position in the x, y or z direction (cm), *BJA*_*(x*,*y*,*z)*_ is the body joint angle in the coronal, transverse or sagittal plane (degrees).

Frame-by-frame analysis was performed to determine at which frame the signal was fluctuating in the same direction, or opposite direction as described previously elsewhere^66^. In other words, for every frame where the position-position or position-angle signal was in agreement (i.e., changing in the same direction), the frame was counted for “same direction” synergy. Conversely, whenever these signals did not correspond (i.e., changing in the opposite direction), the frame was counted for “opposite direction” synergy. The final measure was a % of frames, of total movement element frames, where synergy occurred in the same or opposite directions during the movement element. Equation (3) describes the mathematical procedure to obtain kinematic synergies:

3 $$Synergy\,( \% )=\frac{\#frames\,\{{W}_{vel(x,y)}\times BS{V}_{(x,y,z)}\}\, > \,or\, < 0}{\#\,frames\,(movement\,element\,duration)}\times 100 \% $$

where, Synergy (%) is the kinematic synergy, *W*_*vel(x*,*y)*_ is the wrist instantaneous velocity in the x, limb advance, or y, limb advance or limb drop, direction (cm.s^−1^), *BSV*_*(x*,*y*,*z)*_ is the body segment instantaneous velocity (cm.s^−1^) or instantaneous angular velocity (degrees.s^−1^) in the x, y or z direction.

Synergy graphs throughout the reaching were obtained by summing the number of synergistic events of all animals at each % of the reaching cycle, and expressed as an arbitrary unit (#of counts). Post- and pre-stroke values were subtracted to calculate the difference between pre- and post-stroke kinematic synergy or joint individuation, and this difference was used as input for linear regression and correlation analysis. An overview of the mathematical model developed to automatically compute kinematic synergy or joint individuation using numerical derivatives is described in Supplementary Figure S3. Briefly, as described above, wrist movements were linearly matched to position or angle changes in different segments. Synergies were calculated regarding simultaneous position-position, position-angle or angle-angle (see below) changes and ranged from 0–100%.

Coupling between synergies was calculated using the same algorithm described above. The use of joint angles as main inputs (i.e., elbow flexion and shoulder adduction) instead of wrist movements (movement elements) was the main difference. The coupling index (0–100%) represents the % of angular variation ocurring at the same time (concurrent), e.g., how much adduction occurs during elbow flexion.

### Net trajectory adjustments

For net adjustments in the displacement and velocity profiles, custom algorithms were applied to determine the number of zero crossings in the velocity and acceleration profiles, as described elsewhere^51^. Briefly, the position signal was derived to obtain the velocity profile, the number of zero crossings in this profile is the trajectory adjustment. The velocity signal was then derived to obtain the acceleration profile, the number of zero crossings in this profile represents the velocity adjustment. All the derivation processes were conducted on the raw data (no interpolation) and the edges of the signal were removed after each derivation step, and substituted by the previous/anterior frame (to avoid derivation artifacts). Data were filtered using a 2^nd^ order low-pass Butterworth filter (50 Hz).

### Grip strength

Unilateral grip strength was measured pre- and post-stroke (BIO-GS3, Bioseb, Pinellas Park, USA). Physical restriction of one of the limbs using the index finger, while leaving the othe limb unrestrained, allowed measurement of unilateral grip strength. Five measures for each limb were randomly taken. The highest and lowest values were excluded and the 3 intermediate values were averaged and used for further analysis. Force applied was converted to grams by the apparatus software and then normalized to body weight (g/Kg).

### Statistical analysis

Data are presented as mean ± SEM. Because of the complex nature of the data set, such as varying number of trials, and absence of within subjects trial effects, discrete kinematic movements were compared using linear mixed effects modelling with group (pre- and post-stroke) as the between-subjects variable and percent (of duration through reaching) as the within-subjects variable using an identity matrix variance-covariance structure. When a group by percent interaction was present, Bonferroni-corrected post-hoc tests of the estimated marginal means were used to determine the portion of the reach that differed between groups. All variables were tested for normality using Shapiro-Wilk test followed by paired t-tests to compare between pre- and post-stroke. Holm-Bonferroni was used for familywise correction of multiple paired t-test comparisons. Pearson correlation was used to test for correlation between lesion volume-impairment and several variables (only significant correlations are included). Multiple linear regressions were performed to identify aspects of the functional deficits in pellet retrieval and grip strength following stroke. The following variables were tested for inclusion: elbow flexion synergy, shoulder adduction and extension synergies, ventro-dorsal synergies (composite) and coupling. Detailed model parameters can be found in Supplementary Table 1. All statistical analyses were performed using SPSS v. 17, statistical significance was set at p < 0.05.

### Data availability

All datasets generated during the current study are available from the corresponding author.

## Electronic supplementary material

Supplementary materials

Supplementary Video 1

Supplementary Video 2
