# Supplementary material for: Post-stroke kinematic analysis in rats reveals similar reaching abnormalities as humans
Source: Sci Rep. 2018 Jun 7;8:8738. doi: 10.1038/s41598-018-27101-0 (PMC5992226; doi:10.1038/s41598-018-27101-0)
Supplement: Supplementary file 1 — Supplementary materials [file 41598_2018_27101_MOESM1_ESM.pdf]

Supplementary material

Post-stroke kinematic analysis in rats reveals similar reaching abnormalities as humans

Gustavo Balbinot[1,3], Clarissa Pedrini Schuch[1], Matthew S Jeffers[1,2], Matthew W McDonald[1,2], Jessica M Livingston-Thomas[1,2], Dale Corbett[1,2*]

[1]Department of Cellular and Molecular Medicine, Faculty of Medicine, University of Ottawa, Ottawa, ON, Canada;

[2]Canadian Partnership for Stroke Recovery, University of Ottawa, ON, Canada.

[3]Brain Institute, Federal University of Rio Grande do Norte, Natal, RN, Brazil.

Supplementary figures: 3
Supplementary tables: 2
Supplementary videos: 2

Supplementary material

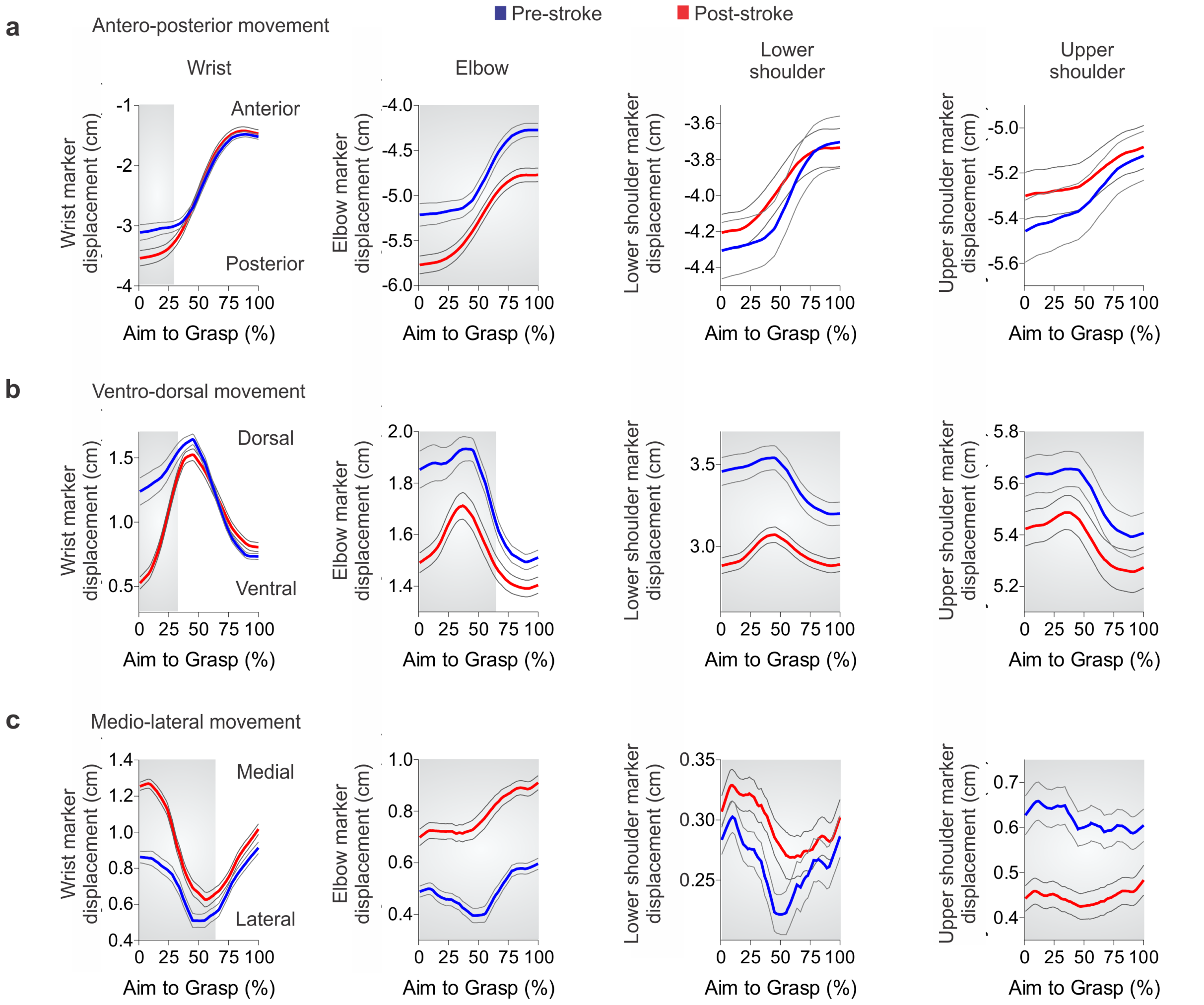

Supplementary Figure S1. Wrist position throughout reaching by plane of movement. Trajectories normalized to a reflective marker placed on the side of step 5. **(a)** In the anterior-posterior plane post-stroke, the wrist joint was further from the target during 1-31% of the reach ($p < 0.001$) and the elbow was also further from the target, relative to pre-stroke, throughout the entire reach ($p < 0.001$). **(b)** In the ventro-dorsal plane the situation was reversed with all joint segments being positioned closer to the target post-stroke. The wrist from 1-32% ($p < 0.001$), elbow from 1-67% ($p = 0.005$) throughout the reach. Lower and upper markers of the shoulder were significantly closer to the target in the ventro-dorsal plane at all points throughout the reach following stroke ($p < 0.001$). **(c)** Joint position in the medio-lateral plane was mixed, with the wrist (1-67%, $p < 0.001$), elbow (1-100%, $p < 0.001$) and lower shoulder (1-100%, $p < 0.001$) being positioned further from the target following stroke. Conversely, the upper shoulder, was positioned closer to the target following stroke throughout the entire reach ($p < 0.001$). All data are mean ± SEM using linear mixed effects modeling with Bonferroni-corrected post-hoc tests. Shaded areas are statistically different between pre- and post-stroke.

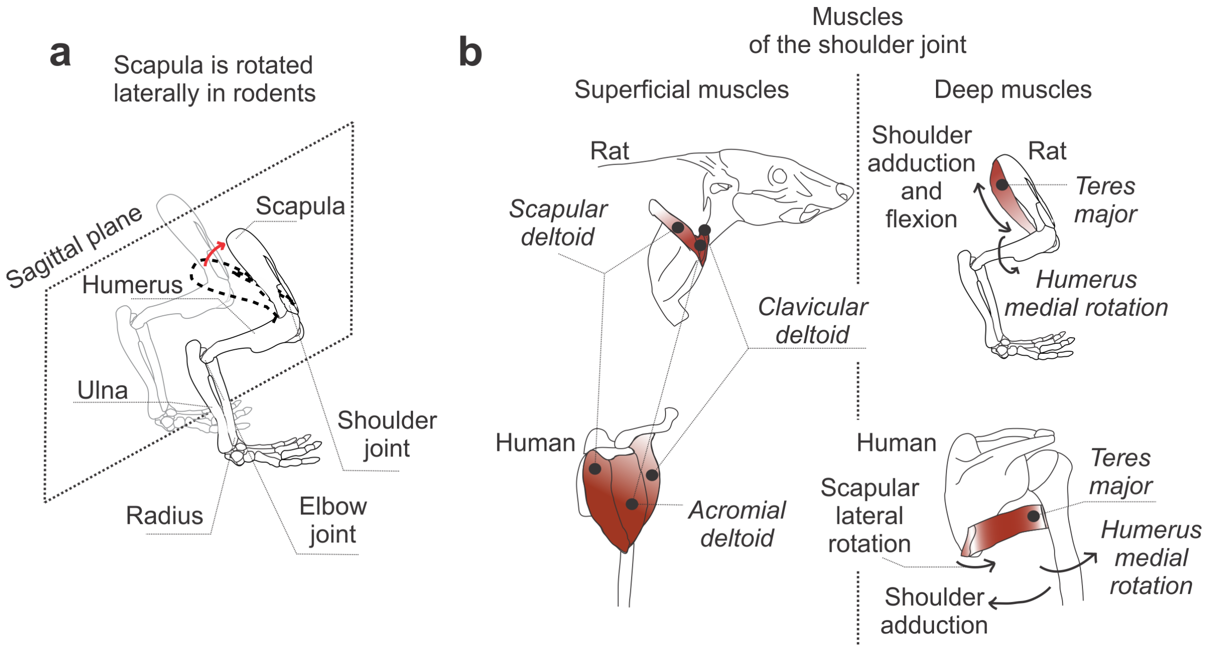

Supplementary Figure S2. Flexion synergy: differences between humans and rodents. (a) In rodents the scapula is rotated into a lateral position (red arrow). (**b**) Muscle mechanics is likely to be affected due to this anatomical change and might explain differences between the biped and quadruped flexion synergy.

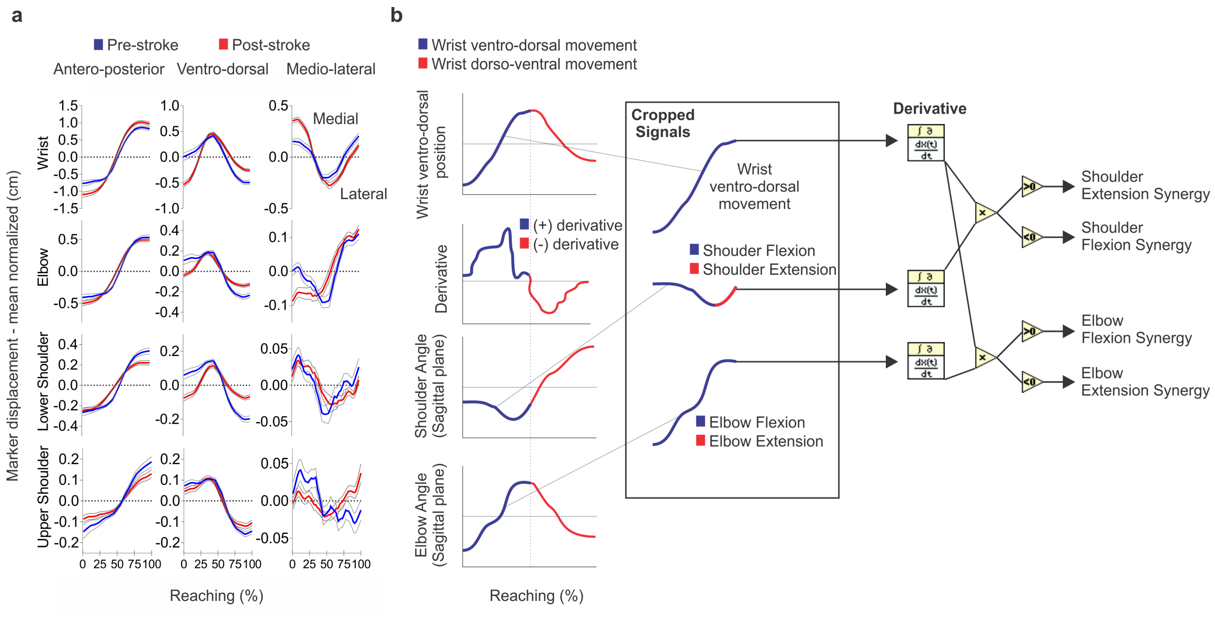

Supplementary Figure S3. Position changes during reaching, kinematic synergy and joint individuation modeling. (**a**) Average and mean subtracted wrist, elbow, lower- and upper-shoulder position changes throughout the reaching (0-100%). (**b**) Model of waveform combinations resulting in kinematic synergies. This schematic diagram illustrates how the kinematic data can be analyzed by linearly combining position-position (joint individuation) or position-angle (kinematic synergy) waveforms. Each of the waveforms has distinct variation across reaching, which produces a unique first derivative patterning. By matching positive and negative signal of first derivatives profiles to position or angle signals (herein exemplified by shoulder and elbow angle variations) it produced a synchronized and linear combination of wrist movement and angle variation. Movement waveforms were then compared using another derivation process, the first derivatives were multiplied by each other, where positive products indicate agreement in variation (i.e., the signals are changing in the same direction) and negative products reflect disagreement in variation (i.e., the signals are changing in opposite direction). The movement waveforms were quantified in terms of synergy ranging from 0-100% of the cropped signal to explain different kinematic synergies. Data are mean ± SEM.

Supplementary Video 1. The emergence of an abnormal elbow flexion synergy hampers the ability to extend the limb and reach for pellets. Example of a severely impaired rat that shows pronounced flexor activity following stroke (from 31.3% ± 4.3% to 67.0% ± 11%; left panel). Note how stroke results in an unsuccessful reach attempt, mainly due to failure to transport the paw to the target. Data are mean ± SEM, n = 1 (left panel), n = 13 (right panel).

Supplementary Video 2. Abnormal movement patterns in rats following stroke. Following stroke rats position the body closer to the target; note that the shoulder is closer to step 5. Rats also perform excessive shoulder and elbow ventro-dorsal movements, resembling shoulder elevation, indicative of a less stable proximal joint. Conversely, pre-stroke movement displays a more stable proximal joint, using elbow and shoulder medio-lateral movements that facilitate paw pronation over the target. Also note the increased elbow flexion at the first half of the reach and decreased extension at the second half of the reach in post-stroke movement.

Supplementary Table 1. Abnormal synergies predictors of change in staircase function and grip strength

Dependent Measure	Predictor	Unstandardized B	95% confidence interval for B		Standardized β	t	p-value
			Lower bound	Upper bound			
Staircase performance (#pellets retrieved; %)	Intercept	43.729	33.473	53.984		9.385	<0.001
	Coupling between shoulder adduction and external rotation (%)	-0.59	-0.107	-0.010	-0.627	-2.666	0.022
Grip strength (%)	Intercept	46.314	32.785	59.843		7.535	<0.001
	Elbow flexion during limb advance synergy (%)	0.074	0.016	0.132	0.649	2.828	0.016

Supplementary Table 2. Summary of upper extremity hemiplegia assessment. Joint movement$_{RMS}$, joint individuation deficit, joint angle$_{RMS}$ and kinematic synergy were measured during limb advance, limb lift and limb drop movements (n = 13). Data are mean ± SEM; *p < 0.05, paired t-test; n.s. = not significant; n.a. = not available; #n = data presented in Figure #n.

**Joint movement$_{RMS}$**				**Joint individuation deficit**			
Joint	Direction	Pre x Post (cm)	Post - Pre (cm)<br>Correlation with lesion volume	Pre x Post (%)<br>Limb advance	 Limb lift	 Limb drop	Post - Pre (%)<br>Correlation with lesion volume
Upper shoulder	Posteriro-anterior	0.139±0.019 (pre)<br>0.107±0.022 (post) n.s.	r = -0.77<br>p = 0.002	Posterior-anterior<br>77.09±4.03 (pre)<br>73.34±4.33 (post)<br>n.s.	Posterior-anterior<br>50.87±5.75 (pre)<br>63.04±4.97 (post) n.s.	Posterior-anterior<br>81.07±3.64 (pre)<br>76.23±4.49 (post) n.s.	r = -0.79 (Limb advance)<br>p = 0.001<br>r = -0.73 (Limb drop)<br>p = 0.004
	Ventro-dorsal	0.102±0.017 (pre)<br>0.111±0.018 (post) n.s.	n.s.	Ventro-dorsal<br>27.24±2.78 (pre)<br>41.27± 3.77(post)*[5a]<br>p = 0.011	Ventro-dorsal<br>66.52±4.06 (pre)<br>69.25±3.52 (post) n.s.	Dorso-ventral<br>78.07±3.02 (pre)<br>71.37±4.58 (post) n.s.	n.s.
	Medio-lateral	0.037±0.007 (pre)<br>0.037±0.007 (post) n.s.	n.s.	Medio-lateral<br>48.25±2.06 (pre)<br>44.53±1.68 (post)<br>n.s.	Medio-lateral<br>47.21±2.97 (pre)<br>51.13±2.74 (post) n.s.	Medio-medial<br>55.83±3.31 (pre)<br>58.33±3.05 (post) n.s.	n.s.
Lower shoulder	Posteriro-anterior	0.246±0.035 (pre)<br>0.215±0.030 (post) n.s.	n.s.	Posterior-anterior<br>86.46±3.48 (pre)<br>84.68±2.38 (post)<br>n.s.	Posterior-anterior<br>49.20± 5.70 (pre)<br>72.47±4.23 (post)*<br>p = 0.004	Posterior-anterior<br>82.84±3.12 (pre)<br>78.12±4.59 (post) n.s.	n.s.
	Ventro-dorsal	0.123±0.013 (pre)<br>0.103±0.021 (post) n.s.	n.s.	Ventro-dorsal<br>30.41±2.64 (pre)<br>47.41±3.46 (post)*[5a]<br>p = 0.001	Ventro-dorsal<br>79.16±3.51 (pre)<br>79.76±2.34 (post) n.s.	Dorso-ventral<br>79.76±2.01 (pre)<br>76.48±3.99 (post) n.s.	r = 0.57 (Limb lift)<br>p = 0.039

	Medio-lateral	0.044±0.008 (pre)<br>0.033±0.010 (post) n.s.	n.s.	Medio-lateral<br>45.01±2.04 (pre)<br>47.25±1.60 (post)<br>n.s.	Medio-lateral<br>53.96±3.60 (pre)<br>51.47±1.70 (post) n.s.	Latero-medial<br>58.51±2.48 (pre)<br>56.62±2.60 (post) n.s.	n.s.
Elbow	Posteriro-anterior	0.400±0.034 (pre)<br>0.408±0.024 (post) n.s.	n.s.	Posterior-anterior<br>92.53±1.13 (pre)<br>94.58±0.96 (post)<br>n.s.	Posterior-anterior<br>48.31±5.17 (pre)<br>76.05±4.35 (post)*<br>$p < 0.001$	Posterior-anterior<br>84.14±2.18 (pre)<br>83.64±4.27 (post) n.s.	n.s.
	Ventro-dorsal	0.167±0.021 (pre)<br>0.147±0.022 (post) n.s.	n.s.	Ventro-dorsal<br>29.15±2.69 (pre)<br>40.99±1.97 (post)*[5a]<br>$p = 0.008$	Ventro-dorsal<br>76.27± 3.55 (pre)<br>77.88±2.92 (post) n.s.	Dorso-ventral<br>77.47±2.57 (pre)<br>78.15±3.63 (post) n.s.	n.s.
	Medio-lateral	0.082±0.013 (pre)<br>0.106±0.013 (post) n.s.	n.s.	Medio-lateral<br>37.43±2.36 (pre)<br>29.98±1.80 (post)*<br>$p = 0.029$	Medio-lateral<br>60.31± 3.38 (pre)<br>45.54±3.96 (post)*<br>$p = 0.001$	Lateral-medial<br>65.09±3.19 (pre)<br>70.86±2.66 (post) n.s.	n.s.
Wrist	Posteriro-anterior	0.684±0.046 (pre)<br>0.871±0.065 (post)*[6c]<br>$p = 0.013$	n.s.	n.a.	n.a.	n.a.	n.a.
	Ventro-dorsal	0.334±0.025 (pre)<br>0.373±0.047 (post) n.s	n.s.	n.a.	n.a.	n.a.	n.a.
	Medio-lateral	0.166±0.022 (pre)<br>0.223±0.026 (post)*[6c]<br>$p = 0.021$	n.s.	n.a.	n.a.	n.a.	n.a.
		**Joint angle$_{RMS}$**		**Kinematic synergy**			
Joint	Plane	Pre x Post (deg.)	Post - Pre (deg.)<br>Correlation with	Pre x Post (%)<br>Limb advance	Limb lift	Limb drop	Post - Pre (%)<br>Correlation with lesion volume

			Lesion volume				
Elbow	Coronal	19.54±3.01 (pre)<br>26.53±3.33 (post)*[4i]<br>p = 0.048*	r = 0.64<br>p = 0.016	External rotation<br>48.46±2.85 (pre)<br>43.49±3.01 (post)<br>n.s.	External rotation<br>40.13±6.30 (pre)<br>40.84±4.51 (post) n.s.	Internal rotation<br>51.56±5.56 (pre)<br>55.75±4.62 (post)*<br>p = 0.028	r = 0.67 (Limb advance/internal rotation)<br>p = 0.011
	Transverse	9.42±1.79 (pre)<br>6.20±0.66 (post) n.s.	n.s.	External rotation<br>36.36±3.16 (pre)<br>39.13±3.39 (post)<br>n.s.	External rotation<br>67.46±4.02 (pre)<br>69.52±3.81 (post) n.s.	Internal rotation<br>69.98±3.26 (pre)<br>81.15±2.24 (post)*<br>p = 0.001	n.s.
	Sagittal	6.63±0.71 (pre)<br>8.00±0.72 (post) n.s.	n.s.	Flexion<br>27.00±2.73 (pre)<br>42.69±3.96 (post)*[4c]<br>p < 0.001	Flexion<br>90.15±1.73 (pre)<br>90.33±1.85 (post) n.s.	Extension<br>92.77±1.66 (pre)<br>94.64±1.27 (post) n.s.	n.s.
Shoulder	Coronal	5.08±1.17 (pre)<br>4.58±0.85 (post) n.s.	n.s.	Adduction<br>51.30±4.22 (pre)<br>67.03±2.90 (post)*<br>p < 0.001	Adduction<br>45.39±3.53 (pre)<br>51.10±4.96 (post)*<br>p = 0.034**	Adduction<br>54.54±4.21 (pre)<br>66.03±3.69 (post)*<br>p = 0.010	n.s.
	Transverse	11.20±2.33 (pre)<br>8.86±1.75 (post) n.s.	n.s.	Adduction<br>66.61±2.51 (pre)<br>74.85±2.54 (post)*<br>p = 0.004	Adduction<br>37.54±2.93 (pre)<br>60.45±4.58 (post)*[4f]<br>p < 0.001	Adduction<br>67.03±4.23 (pre)<br>74.72±3.83 (post) n.s.	r = 0.64 (Limb advance/adduction)<br>p = 0.018
	Sagittal	3.79±0.70 (pre)<br>3.85±0.52 (post) n.s.	n.s.	Flexion<br>36.54±5.28 (pre)<br>34.30±3.87 (post)<br>n.s.	Extension<br>42.52±7.30 (pre)<br>43.60±6.27 (post) n.s.	Extension<br>63.56±5.27 (pre)<br>74.30±2.44 (post)*[5f]<br>p = 0.047	n.s.
